# Comparison of Time to Next Treatment or Death Between Front‐Line Daratumumab, Lenalidomide, and Dexamethasone (DRd) Versus Bortezomib, Lenalidomide, and Dexamethasone (VRd) Among Transplant‐Ineligible Patients With Multiple Myeloma

**DOI:** 10.1002/cam4.70308

**Published:** 2024-11-01

**Authors:** Doris K. Hansen, Santosh Gautam, Marie‐Hélène Lafeuille, Carmine Rossi, Bronwyn Moore, Anabelle Tardif‐Samson, Philippe Thompson‐Leduc, Alex Z. Fu, Annelore Cortoos, Shuchita Kaila, Rafael Fonseca

**Affiliations:** ^1^ Department of Blood and Marrow Transplant and Cellular Immunotherapy H. Lee Moffitt Cancer Center & Research Institute Tampa Florida USA; ^2^ Janssen Scientific Affairs, LLC Horsham Pennsylvania USA; ^3^ Analysis Group, Inc. Montréal Quebec Canada; ^4^ Division of Hematology and Medical Oncology Mayo Clinic Phoenix Arizona USA

**Keywords:** bortezomib, daratumumab, drug therapy, health care, hematologic diseases, multiple myeloma, observational study, outcome assessment

## Abstract

**Introduction:**

Daratumumab, lenalidomide, and dexamethasone (DRd) and bortezomib, lenalidomide, and dexamethasone (VRd) are the only preferred treatment regimens for patients with transplant‐ineligible (TIE) newly diagnosed multiple myeloma (NDMM). As there are no randomized head‐to‐head studies of DRd versus VRd, this analysis aimed to compare real‐world time‐to‐next‐treatment (TTNT) or death in this population.

**Methods:**

Patients with NDMM who received front‐line (FL) DRd or VRd were identified from the Acentrus database (January 1, 2018 to May 31, 2023). Those with a record of a stem cell transplant or aged < 65 years were excluded to limit analysis to the TIE population. Inverse probability of treatment weighting was used to balance baseline patient characteristics. A doubly robust Cox proportional hazards model was used to compare TTNT or death between cohorts.

**Results:**

A total of 149 and 494 patients who initiated DRd and VRd, respectively, were identified. After weighting (weighted N_DRd_ = 302, weighted N_VRd_ = 341), cohorts had similar baseline characteristics. Of these, 98 (32.4%) DRd and 175 (51.2%) VRd patients either received a subsequent line of therapy or died, with a median TTNT or death of 37.8 months in the DRd cohort and 18.7 months in the VRd cohort (hazard ratio: 0.58, 95% confidence interval: 0.35, 0.81; *p* < 0.001).

**Conclusion:**

Treatment of TIE NDMM patients with DRd led to a significantly longer TTNT or death compared to VRd, evidenced by a 42% risk reduction, supporting the effectiveness of DRd over VRd as FL treatment in this patient population.

## 1 Background

Multiple myeloma (MM) is a hematologic malignancy characterized by the accumulation of neoplastic plasma cells in the bone marrow that produce proteins detectable in both the urine and blood [1]. Though MM remains an incurable cancer, it is associated with a 5‐year relative survival rate of approximately 60% in population‐based studies [2, 3]. The recent introduction of novel therapeutics has improved prognosis for patients, with common treatment options including immunomodulatory agents (thalidomide, lenalidomide, and pomalidomide), proteasome inhibitors (bortezomib and carfilzomib), and monoclonal antibodies (daratumumab and isatuximab) [3, 4, 5, 6]. For patients with newly diagnosed MM (NDMM), induction therapy combined with high‐dose chemotherapy and autologous stem cell transplant (SCT) is the preferred treatment option [7, 8]. However, certain patients are ineligible for SCT due to patient‐specific factors, such as age and comorbidities, and such patients generally have a shorter overall survival (OS) compared with SCT‐eligible patients [9, 10].

Daratumumab, lenalidomide, and dexamethasone (DRd) and bortezomib, lenalidomide, and dexamethasone (VRd) are the only preferred regimens recommended by the NCCN Clinical Practice Guidelines in Oncology (NCCN Guidelines) with NCCN Category 1 evidence for front‐line (FL) treatment of patients with transplant‐ineligible (TIE) NDMM [8, 11, 12]. While VRd is the mainstay standard of care treatment for NDMM based on results of the pivotal SWOG S0777 trial, alternative triplet regimens that introduce daratumumab, a CD38‐targeting monoclonal antibody, at earlier lines have demonstrated promising efficacy [8, 13, 14]. DRd was approved for patients with TIE NDMM in June 2019 following results from the phase III MAIA clinical trial, where DRd was associated with a significantly lower risk of disease progression and death compared to lenalidomide and dexamethasone alone (Rd) [13, 15].

Though one randomized head‐to‐head comparison of DRd and VRd for the treatment of TIE NDMM is ongoing (S2209; NCT05561387) [16], there are currently no clinical trial results directly comparing DRd and VRd available. However, other analyses have been performed showing the potential benefit of DRd over VRd among patients with NDMM. The TAURUS noninterventional multicenter chart‐review study reported a 65% risk reduction in disease progression or death for DRd compared with VRd [17]. In addition, two adjusted indirect comparisons found that patients treated with DRd in the MAIA trial compared to patients treated with VRd in either the SWOG S0777 trial or a real‐world setting led to a 40% and 32% risk reduction in disease progression or death, respectively [18, 19].

To further expand the evidence base around the comparative effectiveness of DRd and VRd therapies, this study aimed to compare time‐to‐next‐treatment (TTNT) or death between patients ≥ 65 years old with NDMM who had not received SCT and were treated with FL DRd or VRd in real‐world clinical practice in the United States. TTNT has been selected since it is a well‐established measure to determine the relative effect between therapies when analyzing real‐world data in the absence of disease progression information in retrospective databases. However, it is important to stress that TTNT should not be considered a direct proxy for PFS as it incorporates many factors that may lead to discontinuation of treatment before a diagnosis of progression per the International Myeloma Working Group (IMWG) criteria and, consequently, often underestimates PFS in the real‐world setting [20].

## 2 Materials and Methods

### 2.1 Data Source

Data were obtained from the Acentrus database, a health system electronic medical record (EMR) utilized by > 100,000 prescribers from multiple centers across several states, including several National Cancer Institute‐designated sites and National Comprehensive Cancer Network members. The Acentrus EMR contains inpatient and outpatient data consisting of patient characteristics (e.g., age, gender, race/ethnicity), providers, visits, diagnoses (thus enabling the reporting of individual comorbidities), clinical characteristics (e.g., laboratory test results, vitals), mortality information, medication orders, and administration information (e.g., National Drug Codes, days of supply, doses). Specifically of interest to this study is Acentrus’ detailed prescription fill data on specialty drugs, including daratumumab and bortezomib, and the recency of available data, which allows for a more comprehensive investigation of the use of novel treatment regimens in real‐world practice. All data from Acentrus are fully de‐identified and comply with the patient requirements of the Health Insurance Portability and Accountability Act (HIPAA). Therefore, no institutional review board exemption was sought.

### 2.2 Study Design and Sample Selection

**FIGURE 1:**
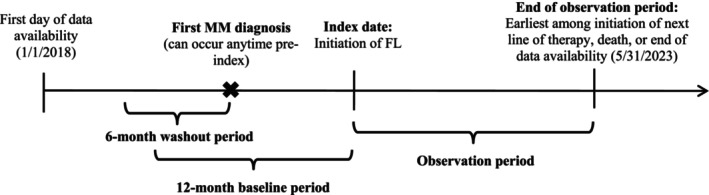
Study design. FL, front‐line; MM, multiple myeloma.

This study used a retrospective, observational cohort design and included Acentrus EMR data from January 1, 2018 to May 31, 2023 (Figure 1). The index date was defined as the initiation of FL DRd or VRd therapy, with ≤ 12 months before the index date constituting the baseline period. Demographic characteristics (i.e., age, sex, race, ethnicity, geographic region, insurance plan type), comorbid conditions, and disease stage were assessed using data from the baseline period or as of the index date. To accurately identify FL therapy for MM, a period of ≥ 6 months without the use of any antineoplastic agents, excluding corticosteroids, prior to the initial MM diagnosis was required (i.e., washout period). The observation period was defined as the time from the index date until the earliest date of initiation of a next line of treatment, death, or end of data availability (May 31, 2023). All medications received within 60 days of the first MM antineoplastic agent were considered part of the FL therapy regimen. The next line of treatment was identified by the initiation of a new antineoplastic agent, excluding corticosteroids, > 60 days following initiation of FL therapy or re‐treatment with the FL regimen after a > 90‐day treatment‐free interval.

**FIGURE 2:**
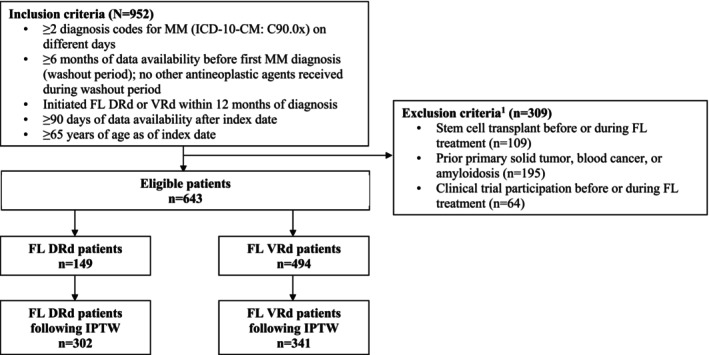
Sample selection. (1) Exclusion criteria were not mutually exclusive. DRd, daratumumab, lenalidomide, and dexamethasone; FL, front‐line; ICD‐10 CM, International Classification of Disease, 10th Revision, Clinical Modification; IPTW, inverse probability of treatment weighting; MM, multiple myeloma; VRd, bortezomib, lenalidomide, and dexamethasone.

Patients were included if they had ≥ 2 records with an International Classification of Diseases, Tenth Revision, Clinical Modification (ICD‐10‐CM) diagnosis code for MM (C90.0x) on different days, including at least one prior to or on the index date, ≥ 6 months of data availability without the use of an antineoplastic agent prior to the first record of MM diagnosis, initiated FL treatment with DRd or VRd within at most 12 months of the first record of MM diagnosis, and ≥ 90 days of data availability post‐index. Patients were excluded if they had a pre‐index diagnosis of amyloidosis or other cancers or participated in a clinical trial before or during FL treatment. To limit the analysis to the TIE population, patients who had a record of an SCT before or during FL treatment and those who were < 65 years old (used as a proxy to SCT eligibility) were excluded (Figure 2) [21].

### 2.3 Measures, Outcomes, and Statistical Analyses

Baseline demographic and clinical characteristics described above were reported separately for patients treated with FL DRd and VRd. In addition to common comorbidities of interest (e.g., anemia, renal impairment), the Quan–Charlson Comorbidity Index (Quan–CCI), which categorizes comorbidities based on ICD diagnosis codes and sums to a single weighted score predicting mortality, was used to quantify the comorbidity burden of patients [22]. Standardized differences were used to assess statistical imbalances between cohorts, where variables with a standardized difference of < 10% were considered balanced [23].

The primary outcome was TTNT or death, defined as the time from the initiation of DRd or VRd to the initiation of the next line of treatment (i.e., initiation of a subsequent different regimen) or death, whichever came first.

Inverse probability of treatment weighting (IPTW) was used to balance patient baseline characteristics between the DRd and VRd cohorts. This method presents the notable advantage of retaining all patients in the analyses, unlike propensity score (PS) matching in which some patients may be removed due to the lack of a suitable match. PSs obtained from a logistic regression model were applied to formulate the IPTW‐derived weights using the following variables: age, gender, race, US region, insurance coverage, index year, International Staging System (ISS) stage, and selected comorbidities (Table 1). For each patient, the IPTW‐derived weight was calculated as 1/PS for patients in the DRd cohort and 1/(1 − PS) for patients in the VRd cohort. All IPTW‐derived weights were normalized by the mean weight, with weights truncated at the 5th and 95th percentile.

**TABLE 1:** Weighted baseline demographic and clinical characteristics^a^ ^,^ ^b^.

	DRd (*N* = 302)	VRd (*N* = 341)	Std. diff. (%)
Age (years) at the index date, mean ± SD [median]	75.3 ± 8.9 [75.0]	74.5 ± 5.0 [74.0]	10.9*
Age ≥ 80 years, *n* (%)	77 (25.5)	74 (21.7)	9.1
Female, *n* (%)	148 (48.9)	156 (45.8)	6.4
Race, *n* (%)			
White	169 (55.8)	200 (58.7)	5.7
Black	26 (8.8)	38 (11.3)	8.4
Asian	7 (2.2)	6 (1.9)	2.5
Other^c^	100 (33.2)	96 (28.2)	10.8*
Ethnicity, *n* (%)			
Non‐Hispanic	189 (62.7)	243 (71.3)	18.6*
Hispanic	24 (7.9)	8 (2.3)	25.7*
Other	5 (1.8)	3 (0.8)	8.5
Unknown	84 (27.6)	87 (25.5)	4.8
US geographic region, *n* (%)			
West	103 (34.0)	111 (32.6)	3.0
South	86 (28.4)	103 (30.2)	3.8
North Central	52 (17.2)	67 (19.8)	6.6
Northeast	24 (8.1)	23 (6.7)	5.3
Unknown	37 (12.2)	37 (10.8)	4.6
Insurance plan type, *n* (%)			
Medicare	190 (62.7)	225 (65.9)	6.8
Managed care	52 (17.2)	67 (19.7)	6.3
Medicaid	5 (1.8)	9 (2.7)	6.2
Other	70 (23.0)	69 (20.3)	6.6
Unknown	11 (3.7)	11 (3.1)	3.5
Index year, *n* (%)			
2019	15 (5.0)	36 (10.6)	21.1*
2020	72 (23.7)	90 (26.5)	6.4
2021	94 (30.9)	104 (30.4)	1.2
2022	98 (32.5)	97 (28.5)	8.7
ISS stage, *n* (%)			
Stage 1	0 (0.0)	2 (0.7)	11.6
Stage 2	23 (7.6)	22 (6.6)	3.9
Stage 3	28 (9.1)	26 (7.7)	5.3
Not available	252 (83.3)	290 (85.1)	4.9
Quan–CCI^d^, mean ± SD [median]	3.8 ± 3.3 [3.0]	3.6 ± 1.9 [2.0]	5.4
Malignancies and metastatic solid tumor, *n* (%)	55 (18.3)	53 (15.5)	7.6
Diabetes without chronic complications, *n* (%)	33 (11.0)	32 (9.4)	5.6
Peripheral vascular disease, *n* (%)	23 (7.6)	31 (9.2)	6.0
Chronic pulmonary disease, *n* (%)	20 (6.6)	30 (8.8)	8.2
Cerebrovascular disease, *n* (%)	17 (5.6)	16 (4.6)	4.8
Rheumatologic disease, *n* (%)	6 (2.0)	6 (1.7)	2.0
Comorbidities of interest, *n* (%)			
≥ 1 CRAB symptom	172 (57.0)	200 (58.6)	3.3
Anemia	113 (37.2)	126 (37.1)	0.3
Skeletal‐related events	89 (29.4)	91 (26.7)	5.9
Renal impairment^e^	36 (12.0)	46 (13.4)	4.4
Hypercalcemia	30 (9.9)	42 (12.3)	7.7
Thyroid disease	33 (11.0)	40 (11.8)	2.5
Secondary malignancy	31 (10.2)	33 (9.6)	2.2
Inflammatory conditions^f^	9 (2.9)	7 (2.1)	5.0

Abbreviations: CCI, Charlson Comorbidity Index; CRAB, calcium elevation, renal insufficiency anemia, and bone abnormalities; DRd, daratumumab, lenalidomide, and dexamethasone; ISS, International Staging System; SD, standard deviation; Std. Diff, standardized difference; US, United States; VRd, bortezomib, lenalidomide, and dexamethasone.

* denotes variables that were included as part of the doubly robust adjustment in the Cox proportional hazards model.

^a^ Inverse probability of treatment weights were estimated using a multivariable logistic regression model with the following baseline covariates: age, gender, race, US region, insurance coverage, index year, ISS stage, selected Quan–CCI comorbidities (shown in Table 1), and additional comorbidities of interest (shown in Table 1, except anemia, skeletal‐related events, and renal impairment).

^b^ The proportions displayed were calculated prior to the rounding and may be slightly different than if they were calculated based on rounded numbers.

^c^ Other race categories included American Indian or Alaska Native, Native Hawaiian or Other Pacific Islander, Multiple Race, Hispanic, and Other.

^d^ Only Quan–CCI comorbidities that were included in the inverse probability of treatment weighting were included in this table.

^e^ Renal impairment was based on diagnostic codes for acute kidney failure or an encounter for care involving renal dialysis.

^f^ Inflammatory conditions included psoriasis, psoriatic arthritis, rheumatoid arthritis, Crohn's disease, ulcerative colitis, ankylosing spondylitis, and systemic lupus erythematosus.

Weighted Kaplan–Meier (KM) curves were used to assess the median TTNT or death for DRd and VRd cohorts. Patients without an event were censored at the end of data availability (i.e., May 31, 2023). A weighted Cox proportional hazards model was used to generate hazard ratios (HRs) comparing TTNT or death between DRd and VRd cohorts. To account for variables that remained imbalanced after IPTW, the weighted Cox model was further adjusted for the following variables (i.e., doubly robust approach): continuous age, other race, ethnicity, and index year. Nonparametric bootstrap procedures with 500 replications were used to calculate 95% confidence intervals (CIs) and *p*‐values for the Cox proportional hazards model.

## 3 Results

After applying the selection criteria, 149 and 494 patients were identified in the DRd and VRd cohorts, respectively (Figure 2). Following IPTW (weighted N_DRd_ = 302, weighted N_VRd_ = 341), baseline characteristics in both cohorts were generally similar, including female sex (DRd: 48.9%, VRd: 45.8%), White race (DRd: 55.8%, VRd: 58.7%), Black race (DRd: 8.8%, VRd: 11.3%), Medicare insurance coverage (DRd: 62.7%, VRd: 65.9%), and mean Quan–CCI (DRd: 3.8, VRd: 3.6; Table 1). Minor imbalances remained for mean age (DRd: 75.3 years, VRd: 74.5 years; standardized difference = 10.9%), other race (DRd: 33.2%, VRd: 28.2%; standardized difference = 10.8%), Hispanic ethnicity (DRd: 7.9%, VRd: 2.3%; standardized difference = 25.7%), non‐Hispanic ethnicity (DRd: 62.7%, VRd: 71.3%; standardized difference = 18.6%), and 2019 index year (DRd: 5.0%, VRd: 10.6%; standardized difference = 21.1%). These were further adjusted for in the doubly robust weighted Cox proportional hazards model.

**FIGURE 3:**
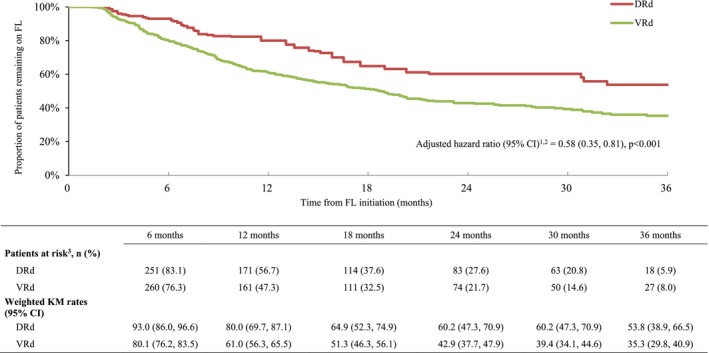
Weighted KM curves for FL time to next treatment or death. (1) In addition to the weighted adjustment, Cox proportional hazards model was further adjusted for age, race, ethnicity, and index year. (2) Non‐parametric bootstrap procedures with 500 replications were used to calculate 95% CIs and *p*‐values. (3) The proportions displayed were calculated prior to the rounding and may be slightly different than if they were calculated based on rounded numbers. CI, confidence interval; DRd, daratumumab, lenalidomide, and dexamethasone; FL, front‐line; KM, Kaplan–Meier; VRd, bortezomib, lenalidomide, and dexamethasone.

The median observation period was 20.2 months for patients treated with DRd and 21.5 months for patients treated with VRd. For patients treated with DRd, the observed median time on FL treatment was 8.2 months, with a median of 6.5 months of active daratumumab use (prior to accounting for censoring). For patients treated with VRd, the observed median time on FL treatment was 5.9 months, with a median of 4.2 months of active bortezomib use (similarly, prior to accounting for censoring). A total of 98 (32.4%) patients treated with DRd and 175 (51.2%) patients treated with VRd received a subsequent line of therapy or died. KM estimates for patients remaining on FL therapy were significantly higher for DRd than VRd at 6 months (93.0% vs. 80.1%; *p* = 0.003), 12 months (80.0% vs. 61.0%; *p* = 0.001), 18 months (64.9% vs. 51.3%; *p* = 0.006), 24 months (60.2% vs. 42.9%; *p* = 0.002), 30 months (60.2% vs. 39.4%; *p* = 0.001), and 36 months (53.8% vs. 35.3%; *p* = 0.002). The median TTNT or death was 37.8 months in the DRd cohort compared to 18.7 months in the VRd cohort. Over the entire observation period, the HR comparing TTNT or death between patients treated with DRd and VRd was 0.58 (95% CI: 0.35, 0.81; *p* < 0.001; Figure 3), indicating a 42% risk reduction of advancing to next treatment or dying for patients treated with FL DRd compared to patients treated with FL VRd.

## 4 Discussion

Results of the current study indicate that patients with TIE NDMM treated with FL DRd in real‐world clinical practice in the United States were 42% less likely to initiate a next treatment or die during the observation period compared to patients treated with FL VRd. To date, no randomized head‐to‐head comparison of DRd and VRd has been completed in patients with TIE NDMM. In addition to the present analysis, only the TAURUS study has directly compared DRd and VRd in the TIE NDMM population using real‐world data, performing a retrospective analysis of medical charts from six academic and three community‐based oncology sites across the United States [17]. Data from the TAURUS study showed that the risk of disease progression or death was 65% lower in patients receiving FL treatment with DRd compared to VRd [17]. Thus, both the current study and the TAURUS results indicate that DRd is more effective than VRd, though the benefit of DRd cannot be directly compared between the two studies as different outcome measures were used.

Indirect analyses using randomized clinical trial (RCT) data have also supported the benefits of DRd over VRd as FL treatment for TIE NDMM. In the PEGASUS study, comparison of patients treated with FL DRd in the MAIA trial versus EMR data of patients treated with FL VRd in a real‐world setting was performed, indicating a 32% lower risk of disease progression or death following FL DRd treatment [19].

Another indirect adjusted analysis was performed using patient‐level data and harmonized inclusion criteria for participants who received either DRd in the MAIA trial or VRd in the SWOG S0777 trial, showing a 40% lower risk of disease progression or death following DRd treatment [18]. Due to differences in the enrollment criteria of both studies (the MAIA trial included only patients with TIE NDMM while the SWOG S0777 trial enrolled a mixed NDMM population including patients who were eligible for SCT but chose to defer or refuse treatment [13, 14]), that particular analysis [18] as well as other subsequent analyses have included only a portion of SWOG S0777 trial participants to improve the relevance of findings to TIE NDMM [24, 25, 26].

Relative improvements in PFS with DRd treatment have also been noted by network meta‐analyses, including a comparison of 23 unique treatment regimens for TIE NDMM using longer follow‐up data from the MAIA and SWOG S0777, in which DRd was found to have the highest probability of prolonging both PFS and OS compared to all other treatment options [24, 26, 27, 28, 29]. In addition to superior outcomes, DRd was shown to provide the best balance between safety and efficacy [25].

Recent findings from the IMROZ and BENEFIT trials suggest that VRd‐based quadruplet regimens result in superior clinical outcomes compared to triplet regimens in patients with TIE NDMM [30, 31]. In the IMROZ trial, isatuximab (Isa) in combination with VRd (Isa‐VRd) was compared to VRd alone in patients with TIE NDMM aged ≤ 80 years [30]. Similarly, the BENEFIT trial, a non‐registrational study, evaluated Isa‐VRd versus Isa‐Rd in patients with TIE NDMM aged ≤ 79 years who were not frail [31]. Patients treated with Isa‐VRd versus VRd in the IMROZ trial had a 40% reduction in the risk of disease progression or death after a median 5‐year follow‐up, while those treated with Isa‐VRd versus Isa‐Rd in the BENEFIT trial had a significantly improved minimal residual disease (MRD) 10^−5^ negativity rate at 18 months in favor of Isa‐VRd. However, it is important to note that the patient population in both the IMROZ and BENEFIT trials differ significantly from the patients enrolled in the MAIA trial. As stated above, both the IMROZ and BENEFIT trials excluded patients older than 80 years, while approximately 13% of the MAIA trial cohort were over 80 years old. Furthermore, the MAIA population was considerably frailer compared to the IMROZ and BENEFIT populations, as 17% of patients treated with VRd in the MAIA trial had an Eastern Cooperative Oncology Group Performance Status (ECOG PS) of ≥ 2, compared to 10% in the IMROZ trial, while the BENEFIT trial excluded frail patients altogether. The efficacy of DRd in frail patients has been evaluated in an earlier subgroup analysis by Facon et al., whereby 172 of 368 (46.7%) patients treated with DRd and 169 of 369 (45.8%) patients treated with Rd were classified as frail using a simplified frailty score based on patient age, CCI, and ECOG PS [32]. These data indicated that after a median follow‐up of 36.4 months, DRd treatment resulted in a 38% reduction in the risk of disease progression or death compared to Rd alone in this frail population.

While the IMROZ and BENEFIT trials demonstrate the potential benefits of anti‐CD38‐based quadruplet therapy, it is also critical to consider the treatment‐related side effects. Notably, the frequency of grade ≥ 3 peripheral neuropathy was considerably lower in the DRd group (2.5%) in the MAIA trial compared to the Isa‐VRd group (7.2%) in the IMROZ trial, or the Isa‐VRd (27.4%) or Isa‐Rd (9.6%) groups in the BENEFIT trial. Given that peripheral neuropathy, a known side effect of bortezomib, can lead to significant disabilities, it is essential to carefully balance efficacy and tolerability when selecting the optimal treatment strategy for older and particularly frailer patients. Per the data of the MAIA study, DRd has proven to provide a significant PFS even in the frailer patient population [32] and has a proven OS benefit in the TIE patient population with a proven acceptable long‐term tolerability profile [33]. Nevertheless, further research is needed to evaluate real‐world effectiveness and to determine the most appropriate treatment regimens for TIE NDMM patients.

The ongoing randomized, phase III S2209 trial (NCT05561387) will be the first head‐to‐head comparison of DRd and VRd in frail or a subset of intermediate fit (CrCl < 30 mL/min, occurrence of cytopenias, or Revised‐ISS [R‐ISS] stage III disease) patients, as determined by the IMWG frailty score, evaluating DRd versus VRd‐Lite (i.e., VRd at reduced dosing) followed by continued DR or R therapy until disease progression [16]. Thus, the S2209 trial will directly assess the relative effectiveness of DRd versus VRd, as well as evaluate clinical outcomes among a frail population for which quadruplet regimens have not been proposed as the new standard of care.

A notable strength of the current study was that the Acentrus database provides access to recent data allowing to perform this analysis with real‐world data exclusively. RCTs usually enroll a selected group of participants treated under a strict protocol. As a result, findings may not necessarily apply to a real‐world patient population. Real‐world data, on the other hand, provides information on current clinical practice and treatment effectiveness in the population of interest, including frailer and older patients who are usually excluded from clinical trials. As DRd was approved for treatment of TIE NDMM in June 2019, multiple years of retrospective real‐world data comparing DRd to other FL treatments including VRd are now available. Another key strength of the current study lies in the technique employed to balance patient characteristics. Matching on PS allowed to select two samples of patients with similar characteristics at treatment initiation. Moreover, the doubly robust adjustment further mitigated the risk of confounding bias. Taken together, the current findings of this analysis add to the breadth of evidence supporting the effectiveness of DRd over VRd as FL treatment in patients with TIE NDMM.

### 4.1 Limitations

As this study utilized real‐world EMR data, which typically lack or do not include MM‐specific information on disease progression, the comparative effectiveness between treatment regimens was measured using TTNT instead of PFS. TTNT as an absolute measure is a reflection of multiple factors leading to treatment discontinuation (e.g., treatment cost, insurance, tolerability, physician and patient decision) that may precede diagnosis of progression per the IMWG criteria and is therefore not a direct proxy for PFS. In the United States, in particular, initiation of subsequent therapies due to suboptimal response is common. For example, in the GRIFFIN study, several instances of early discontinuation of VRd due to suboptimal efficacy and initiation of salvage therapies prior to actual development of disease progression were observed, which led to the early censoring of these patients [34]. TTNT has been shown to underestimate PFS in real‐world settings due to the initiation of such salvage therapies prior to progression [20]. Therefore, the direct comparison of median PFS to median TTNT, especially in the United States, may not be appropriate. Nonetheless, the relative effectiveness between treatments using either outcome is informative. On this note, the relative effectiveness of DRd observed in the current study (HR = 0.58 using TTNT, *p* < 0.001) was very similar to what was observed in the MAIA study (HR = 0.55 using PFS, *p* < 0.0001) [33]. Furthermore, patients in the VRd cohort may have heterogeneity in the frequency of administration of bortezomib (i.e., used traditional VRd and VRd lite) [35], which was not assessed in this study. Additionally, Acentrus is a provider‐based data source in which records are only available to the extent that visits are part of the Acentrus network. The database does not capture the services patients received from providers outside of the network. This also applies to date‐of‐death information unless it was provided by the Acentrus‐associated health systems. As a result of administrative (right) censoring, it may have been possible to miss SCTs if such procedures occurred after the end of the data cut‐off period. Furthermore, key patient descriptors, including ECOG PS, frailty, and cytogenetic profile, as well as ISS disease stage for the majority of patients, were not available in the database. Subsequently, potential unobserved confounders were not accounted for in the IPTW, though observed imbalances in baseline characteristics between the DRd and VRd cohorts were considered in the IPTW and doubly robust adjustment to the extent possible. Any impact that missing demographic and clinical variables may have had on patient TTNT or death was expected to have similarly affected both treatment cohorts and was unlikely to have resulted in directional bias. Finally, results may not be generalizable to all patients with TIE NDMM treated with DRd. The study only included patients ≥ 65 years old treated within the Acentrus health system network, comprising data from select centers that may not be representative of overall US population demographics, including the Black patient population.

## 5 Conclusions

This retrospective cohort study analyzed EMR data of patients with TIE NDMM and determined that treatment using FL DRd led to a significantly longer TTNT or death compared to FL VRd, evidenced by a 42% risk reduction, supporting the effectiveness of DRd over VRd as FL treatment in patients with TIE NDMM. Given that there are no head‐to‐head trials comparing DRd and VRd treatment, this study provides meaningful evidence to help inform clinician decision‐making.

## Author Contributions

**Doris K. Hansen:** conceptualization (equal), investigation (equal), methodology (equal), writing – original draft (equal), writing – review and editing (equal). **Santosh Gautam:** conceptualization (equal), data curation (equal), funding acquisition (equal), methodology (equal), project administration (equal), resources (equal), supervision (equal), writing – original draft (equal), writing – review and editing (equal). **Marie‐Hélène Lafeuille:** conceptualization (equal), formal analysis (equal), investigation (equal), methodology (equal), project administration (equal), resources (equal), supervision (equal), validation (equal), visualization (equal), writing – original draft (equal), writing – review and editing (equal). **Carmine Rossi:** conceptualization (equal), formal analysis (equal), investigation (equal), methodology (equal), resources (equal), supervision (equal), writing – original draft (equal), writing – review and editing (equal). **Bronwyn Moore:** formal analysis (equal), investigation (equal), software (equal), validation (equal), visualization (equal), writing – original draft (equal), writing – review and editing (equal). **Anabelle Tardif‐Samson:** formal analysis (equal), investigation (equal), software (equal), validation (equal), visualization (equal), writing – original draft (equal), writing – review and editing (equal). **Philippe Thompson‐Leduc:** conceptualization (equal), formal analysis (equal), investigation (equal), methodology (equal), project administration (equal), resources (equal), supervision (equal), validation (equal), visualization (equal), writing – original draft (equal), writing – review and editing (equal). **Alex Z. Fu:** conceptualization (equal), data curation (equal), funding acquisition (equal), methodology (equal), project administration (equal), resources (equal), supervision (equal), writing – original draft (equal), writing – review and editing (equal). **Annelore Cortoos:** conceptualization (equal), data curation (equal), funding acquisition (equal), methodology (equal), project administration (equal), resources (equal), supervision (equal), writing – original draft (equal), writing – review and editing (equal). **Shuchita Kaila:** conceptualization (equal), data curation (equal), funding acquisition (equal), methodology (equal), project administration (equal), resources (equal), supervision (equal), writing – original draft (equal), writing – review and editing (equal). **Rafael Fonseca:** conceptualization (equal), investigation (equal), methodology (equal), writing – original draft (equal), writing – review and editing (equal).

## Ethics Statement

Data were de‐identified and complied with the patient requirements of the Health Insurance Portability and Accountability Act (HIPAA) of 1996; therefore, no review by an institutional review board was required per Title 45 of CFR, Part 46.101(b) (4).

## Conflicts of Interest

Doris K. Hansen reports research funding from Bristol‐Myers Squibb, Karyopharm, and Adaptive Biotech, as well as a consulting or advisory role for Bristol‐Myers Squibb, Janssen, Pfizer, Kite, and Karyopharm. Dr. Hansen is supported by the Pentecost Family Myeloma Research Center. Santosh Gautam, Alex Fu, Annelore Cortoos, and Shuchita Kaila are employees of Janssen Scientific Affairs, LLC, and may own Johnson & Johnson stock/stock options. Marie‐Hélène Lafeuille, Carmine Rossi, Bronwyn Moore, Anabelle Tardif‐Samson, and Philippe Thompson‐Leduc are employees of Analysis Group, Inc., a consulting company that has provided paid consulting services to Janssen Scientific Affairs, LLC, which funded the development and conduct of this study and manuscript. Rafael Fonseca reports a consulting or advisory role for AbbVie, Adaptive, Amgen, Apple, Bristol‐Myers Squibb/Celgene, Caris Life Sciences, GSK, Janssen, Karyopharm, Pfizer, RA Capital, Regeneron and Sanofi. Dr. Fonseca also sits on the board of directors of Antenge and holds a patent for fluorescence in situ hybridization in multiple myeloma.

## Previous Presentation

Part of the material in this manuscript was presented at the American Society of Hematology conference held December 9–12, 2023, in San Diego, CA, USA as an oral presentation.

## Data Availability

The data that support the findings of this study are available from Acentrus. Restrictions apply to the availability of these data, which were used under license for this study.
